# Hotspotters Project: a study protocol for a stepped wedge cluster RCT on the cost-effectiveness of 12-month proactive, integrated and personalised care for patients with problems on multiple life domains and high acute care use

**DOI:** 10.1136/bmjopen-2024-087940

**Published:** 2025-08-10

**Authors:** Vera Tiemes, Kimberley A Leming, Hanneke Borgdorff, Marc Abraham Bruijnzeels, Laurens C van Gestel, Marieke A Adriaanse, Mark van der Wel, Elske M van den Akker-van Marle, Mattijs E Numans, Rimke C Vos

**Affiliations:** 1 Department of Public Health and Primary Care/Health Campus the Hague, Leiden University Medical Center (LUMC), Leiden, The Netherlands; 2 Department of Health, Medical and Neuropsychology, Universiteit Leiden, Leiden, Netherlands; 3 Radboud Institute of Health Sciences, Department of Primary and Community Care, Radboud University Medical Center, Nijmegen, Gelderland, Netherlands; 4 Biomedical Data Sciences, Leiden University Medical Center, Leiden, Netherlands

**Keywords:** Primary Health Care, Health Care Costs, Delivery of Health Care, Integrated, Patient Care Management, Patient-Centered Care

## Abstract

**Introduction:**

‘Hotspotters’ are patients with complex care needs, defined by problems in multiple life domains and high acute care use. These patients often receive mismatched care, resulting in overuse of care and increased healthcare costs. As reliable data on effective interventions for this population are scarce, the goal of this study is to assess the cost-effectiveness of proactive, personalised, integrated care for this group.

**Methods and analysis:**

The Hotspotters Project is planned as a stepped wedge cluster randomised controlled trial in 20 primary care practices in the Netherlands. All practices and participants will begin with standard care during the control period (2–8 months), followed by an intervention (12 months) consisting of a positive health intake with goal setting, multidisciplinary meetings, a personalised care plan and proactive care management. The study will conclude with a follow-up (2–8 months), resulting in a total study duration of 22 months. We plan to include 200 patients with (a) problems on two or more life domains and (b) at least two acute care encounters in the previous year. Possible Hotspotters are identified using an Adjusted Clinical Groups-based algorithm or via a local primary healthcare team.

**Outcomes:**

Questionnaires and routine care data will be used to gather data on cost-effectiveness, which will then be assessed using multilevel analysis, with levels for the individual, cluster and duration of control period. Secondary outcomes will include psychological outcomes on self-regulation (proactive coping, patient activation, self-efficacy and intention), experience of care (satisfaction, perceived autonomy support and qualitative data from focus groups) and quality of life, qualitative analysis of the Positive Health approach, implementation outcomes and process evaluation including integration of care.

**Ethics and dissemination:**

The Ethics Committee of Leiden University Medical Centre granted approval (METC-LDD, P21.123). Results will be shared through peer-reviewed publication and (inter)national conference presentations.

**Trial registration number:**

NCT05878054.

**STRENGTHS AND LIMITATIONS OF THIS STUDY**

- The stepped wedge design, which ensures all participants first take part in the control phase followed by the intervention, will improve control data and reduce the effect of regression to the mean.
- All communication is tailored for low literacy, and telephone support is offered for written questionnaires.
- The intervention involves professionals from medical, mental and social domains within the general practitioner practice network, which is already established or easily formed locally, supporting long-term implementation.
- A limitation of the stepped wedge approach is the potential for increased dropout.
- The evolving ‘Hotspotters’ definition limits comparability with earlier studies targeting similar high-need patients.

## Introduction

 This study will test a novel intervention aimed at ‘Hotspotters’—patients with health problems spanning physical, mental and/or social domains, in combination with high acute care use. The definition of this population is based on Gawande’s landmark article on ‘Hot Spotting’,^1^ which described that accumulation of medical problems concentrates geographically—in a neighbourhood, street or even a building. This article resulted in increased attention from researchers and policymakers for patient groups based on costs or use of care, previously referred to as high-cost patients,^2^,^6^ high-cost high-need patients,^7^,^13^ super-utilisers of care and frequent attenders of emergency departments (EDs).^14^,^22^ The extensive use of (acute) care suggests that existing care options do not match Hotspotters’ care needs. This results in costly^23 24^ and ineffective care, suboptimal health outcomes and a poor patient experience of care.^17^ In addition to a heavy but partially avoidable burden on the healthcare system as a whole,^3 4 18 23^ healthcare professionals may become frustrated and feel they are ‘fighting a losing battle’.^1^

This current study defines Hotspotters by disturbed life domains (a needs-based perspective) and high acute care use (current care needs, consequence on care system). Problems on multiple life domains will result in various coexisting conditions, each impacting daily life. Physical problems refer to chronic physical conditions, and mental problems to diagnosis such as depression or substance abuse. By also including problems in the social domain (eg, housing, poverty, loneliness), we build on the ‘Hot Spotting’ concept where the geographical clustering points to sociodemographics as a contributing factor. The co-occurrence of high acute care use and problems in multiple life domains is poorly documented, but prevalence has been estimated at 0.9%.^25^ Better recognition and care for this patient group, especially proactive or preventive interventions, may help spur better performance across the entire health system.

Due to their high disease burden and high care use, care interventions for Hotspotters offer substantial room for improvement. For instance, a pilot study in the USA found that spending on patients with a chronic condition, mental health issues and substance abuse was at least four times higher than for the chronically ill without the latter problems. An intervention by health resilience workers substantially reduced the annual mean number of ED visits (9.3 to 6.2) and hospital admissions (2.0 to 1.3).^26^ This led to our first pilot, a case series assessing whether positive health interview could be considered suitable care for the problems that Hotspotters face. Twentyfive patients with high acute care use and problems on multiple life domains were identified and recruited by their general practitioner (GP). In the intervention, patients received an extensive positive health interview^27 28^ by their care provider, followed by welfare support tailored to individual needs such as housing or finding volunteer work. The mean positive health score improved by 22%, from 5.5 (1.5 SD) to 6.7 (1.1 SD), at 1 year (*unpublished*).

Hotspotters’ transdisciplinary problems are often addressed ad hoc and without coordination, whereas Hotspotters would likely benefit from a non-standard, integrated approach to social, mental and physical problems. Intervention studies aimed at patients with complex care needs vary greatly in terms of population, content and location of care^8 15 29^ and have produced mixed results regarding care utilisation, clinical and patient-reported outcomes.^8 9 11 15 20 22 29^ However, due to observational data characterised by a ‘regression to the mean effect’ in a group with extreme healthcare utilisation, these studies often face methodological design issues, complicating interpretation of intervention effects even in randomised controlled trials (RCTs).^20^,^22^ The variety of reported interventions adds further complexity, but interventions based on case or care management, integrated or personalised care have shown promising results.^8 20 30^

Our second pilot was a feasibility study for the current intervention study, which included the selection of patients using an algorithm, randomisation, invitation for study participation using standard study information leaflets and central recruitment (*unpublished*).^31^ This pilot showed that the study protocol was feasible and highlighted recruitment considerations which have been incorporated into the current study. Its lessons are reflected in the current design and implementation. Building on our two pilot studies, we now aim to evaluate the cost-effectiveness of a 12-month proactive, personalised care approach for Hotspotters that integrates physical, mental and social care. This primary care-based intervention will consist of a positive health interview^27 28^ and goal setting, multidisciplinary meetings, a personalised care plan and proactive care management. In a Triple Aim approach, we hope to simultaneously improve the experience, reduce the cost of care per capita and improve population health.^32^ Secondary outcomes aim for a better understanding of the mechanisms underlying this complex intervention and include psychological outcomes on self-regulation (proactive coping, patient activation, self-efficacy and intention), experience of care (satisfaction, perceived autonomy support and qualitative data from focus groups), quality of life and qualitative analysis of a positive health approach. Implementation outcomes and process evaluation will provide insight for future implementation.

## Methods

This study protocol is reported in accordance with the *Standard Protocol Items: Recommendations for Intervention Trials* (SPIRIT) reporting guidelines.^33^

### Study design

**Figure 1:**
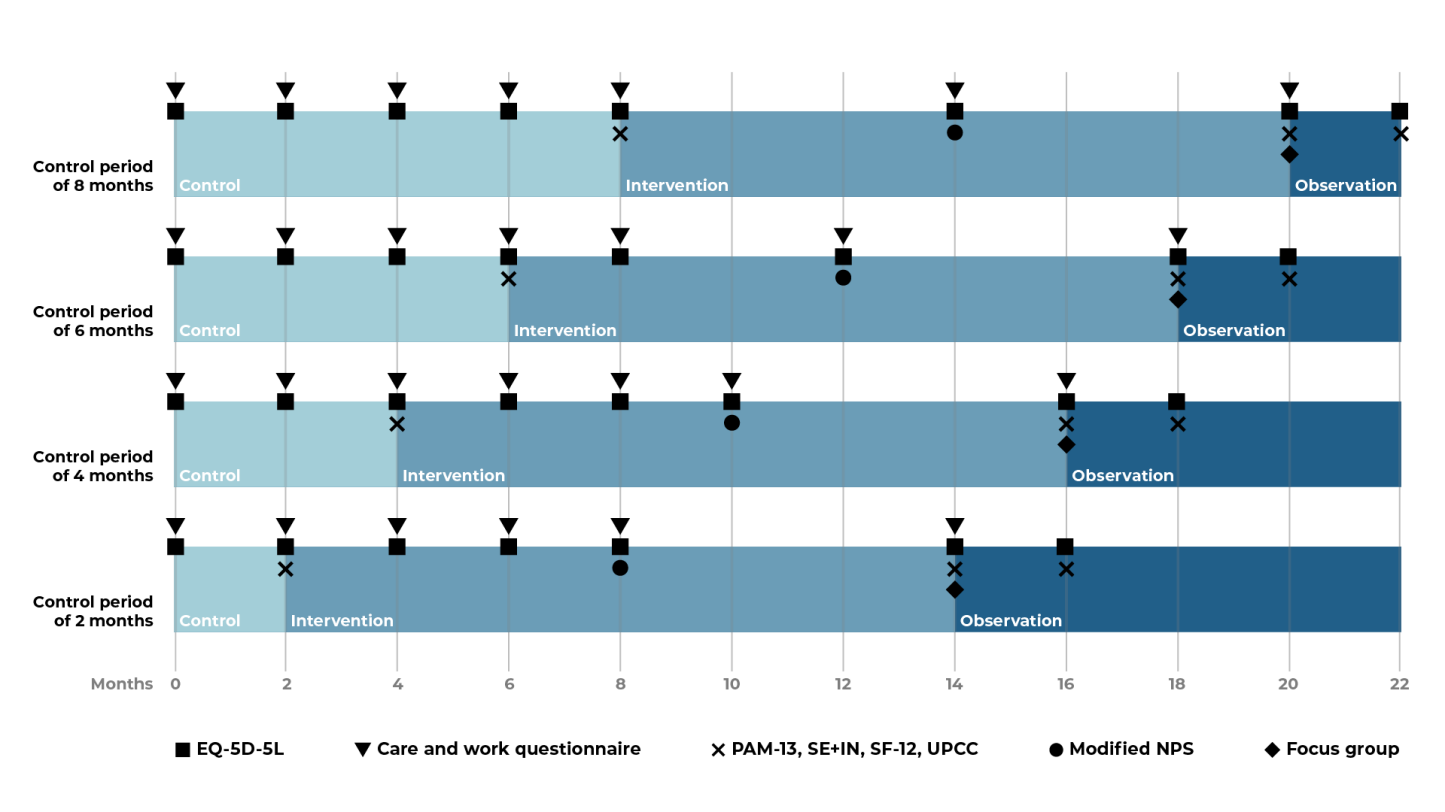
Study design and timing of questionnaire: Primary care practices are randomised to start after a control period of 2, 4, 6 or 8 months. The questionnaires are timed accordingly. EQ-5D-5L, European Quality of Life 5 Dimensions 5 Level Version; NPS, Net Promoter Scale; PAM-13, Patient Activation Measure 13; SE+IN, self-efficacy and intention; SF-12, Short Form Health Survey; UPCC, Utrecht Proactive Coping Competence scale.

This study uses a stepped wedge cluster RCT design, which involves random and sequential crossover of clusters from control to intervention until all clusters are exposed to the intervention.^34^ Clusters—as defined by a team of primary care physician(s) and a mental healthcare nurse cooperating in the same practice population—will be randomised to determine the duration of the control period (figure 1). Randomisation will be performed centrally by two researchers (VT and RCV) using computer-generated random numbers and will be announced after recruitment. Total study duration is 22 months, consisting of a variable control period (2–8 months), an intervention period (12 months) and variable follow-up (2–8 months) (figure 1). We aim to include 20 clusters with a total of 200 participants.

### Sample size calculation

Study sample size is determined by multiplying the unadjusted sample size for cost-effectiveness with the design effect according to a power analysis for stepped wedge cluster RCTs.^35^ The unadjusted sample size for a two-armed RCT was estimated as 380, with 190 cases per arm, based on an average 1-year Health Insurance Act spending of a mean €22 494 (€24 585 SD),^36^ with 80% power, 5% alpha, two-sided equality and a 25% decrease in costs. This power analysis is based on one baseline measure, four steps, intracluster correlation (ICC) of 0.1, 10 patients per cluster and a loss to follow-up of 20%. The ICC was estimated at between 0.01 and 0.3,^37^ but to ensure adequate power, an ICC of 0.1 was chosen as this results in the highest sample size. This yields a design effect of 0.54, leading to a sample size of 204 patients equally distributed over 20 clusters (10 per cluster).

### Study population

All inclusion/exclusion criteria must be met before patients are invited by the practices. Two weeks after study information is sent out, the researcher or research support centre calls for informed consent. Study participation starts after written informed consent (see online supplemental file S1). Possible Hotspotters will be selected using two methods:

- The validated Adjusted Clinical Groups (ACG) method, to identify patients with multimorbidity,^38 39^ will be combined with an algorithm that provides a risk score for each patient that predicts problems in multiple life domains and high acute care use.^25^ This ACG-based algorithm analyses data from primary care electronic health records (EHRs) using sex, age, the International Classification of Primary Care (ICPC) and Anatomical Therapeutic Chemical (ATC) codes, as well as the number of recent primary care consultations. A trusted third party communicates the de-pseudonymised results to the practices.
- Local primary care staff will prepare a list of potential Hotspotters. If available, a list of frequent attenders will be provided to help the team identify other potential Hotspotters.

The 60 patients with the highest algorithm score and all patients identified by practices will be assessed against inclusion and exclusion criteria by primary care staff. Recruitment will alternate between practice and algorithm lists to ensure a 50/50 split, and patients found on both lists will be first approached. The internal order of the lists will be randomised using computer-generated random numbers and per-cluster inclusion ends when no more patients are available or when the cluster reaches their maximum number of inclusions (with a minimum of 10). The selection method has no impact on the intervention and is therefore not discussed with the participants. The study started with its first enrolment in August 2023.

### Inclusion and exclusion criteria

A patient must meet four inclusion criteria to qualify for participation: 18 years and older, registered with the participating practice, a minimum of two acute care encounters in the previous year and problems in two or more life domains (social, mental or physical). Acute care encounters are defined as contact with an out-of-office-hours primary care service, ED, unexpected hospitalisation or contact with acute mental healthcare services. The ICPC and ATC codes will be used to identify registered life domain problems. In Dutch primary care, all contacts are registered in the EHR in an episode list—a complete overview of a patient’s health problems—containing a title formulated by the physician and an ICPC code. Important health problems can be marked as problematic and so remain at the top of the episode list. We define a physical problem when at least one physical ICPC code is flagged as problematic on the episode list; a mental health issue as at least one ICPC code from the ‘P’ chapter either flagged problematic on the episode list or as a contact reason, or if medication related to mental health issues was prescribed; a social problem by having at least one ICPC code from the ‘Z’ chapter on the episode list or as a contact reason.

A physician may exclude a patient from participation for the following reasons: terminally ill, has dementia or cannot communicate effectively, is not competent to make health-related decisions, lives in a residential home, has experience with the conversation tool used in the intervention or veto by the physician for some other reason.

### Control period: care as usual

The control period consists of usual care. Care providers are not encouraged to work proactively together, and consultation is at the patients’ initiative.

### Formal training

Mental healthcare nurses are required to be formally trained in the use of a semi-structured conversation tool on perceived health in multiple domains. Therefore, we offer formal training in positive health methodology.^27^ If they are already trained in using the 4D model, this may be used instead.^40^

### Intervention: personalised, integrated and proactive care

**Figure 2:**
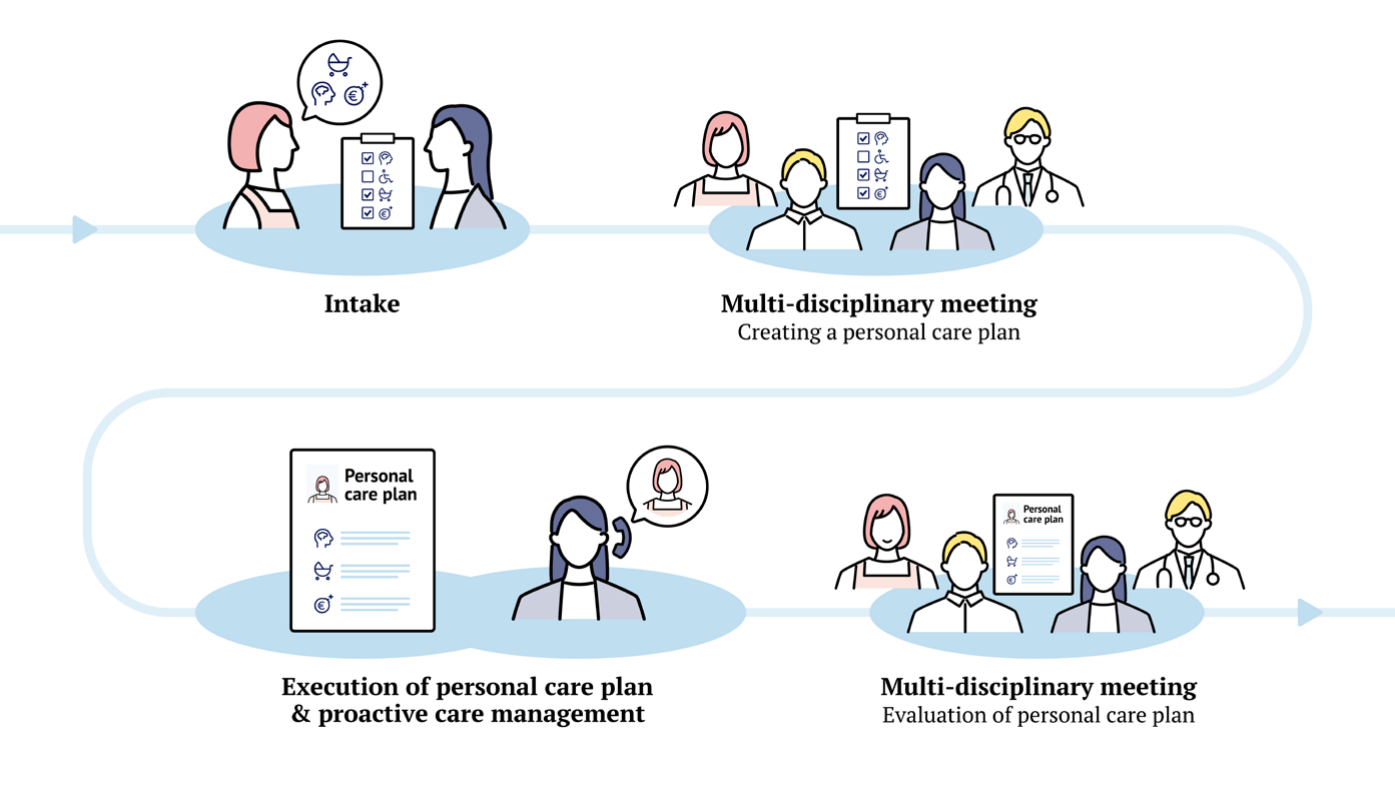
Patient journey: the intervention starts with an intake consultation on perceived health in multiple domains, as well as goal setting. A personalised care plan is then made during a multidisciplinary meeting with the physician, mental healthcare nurse and social worker. Patient participation is encouraged. Execution of the care plan is supported by proactive care management, and progress is evaluated in a second meeting.

The intervention consists of a positive health or 4D intake with goal setting, followed by multidisciplinary meetings, a personalised care plan and proactive care management (figure 2). The first step is a 1-hour intake consultation in which the patient and mental healthcare nurse address all life domains using the semi-structured conversation tool to provide both patient and care provider with perspective on the patient’s needs across domains. A multidisciplinary meeting, including the physician, mental healthcare nurse and social worker, is then held to create a personalised care plan and assign a care coordinator. Patient participation in this meeting is encouraged. The care coordinator supports care plan execution and proactively maintains contact with the patient. The extent and nature of this contact are tailored to the patient’s needs but consist of at least four contact moments, three of which occur in the first three months. Progress and possible care plan adaptation are assessed during a second multidisciplinary meeting, with additional ad hoc consultations or multidisciplinary meetings planned as necessary.

### Observation period: care as usual

Participants return to care as usual when the 1-year intervention ends. During this observation period, study measurements continue to allow evaluation of sustained intervention effects.

### Measurements

All written questionnaires will be sent per email or post, or may be administered per phone interview either on request or when a questionnaire is incomplete. Data from questionnaires will be securely stored in EDC Castor. An overview of all outcomes can also be found in table 1, describing per outcome the measurement instrument(s) used, number of items per instrument, frequency and method of administration. The timing of questionnaires depends on randomisation and is shown in figure 1.

**Table 1:** An overview of the measures used per outcome, the frequency and method of administration

Outcome	Measurement instrument	Items per instrument	Method of administration	Interval (frequency)
Cost-effectiveness	Quality of life, measured by EQ-5D-5L, (European Quality of Life 5 Dimensions 5 Level)^41 42^	6	Written questionnaire for participants*	Every 2 months (7 or 8)
	Cost of care, measured by Care and Work questionnaire *Selected items of IMTA productivity costs questionnaire (iPCQ),*^*43 44*^* IMTA medical consumption questionnaire (iMCQ)*^*45*^*and TiC-P, a questionnaire on healthcare consumption and productivity losses for patients with psychiatric disorders*^*46*^	8	Written questionnaire for participants*	Every 2 months (7 or 8)
Self-regulation	Utrecht Proactive Coping Competence scale^48^	21	Oral administration of questionnaire with participant	At baseline, after the interventions and after 2 months follow-up (3)
	Patient Activation Measure 13^49^	13	Oral administration of questionnaire with participant	At baseline, after the interventions and after 2 months follow-up (3)
	Self-efficacy and intention item list	8	Oral administration of questionnaire with participant	At baseline, after the interventions and after 2 months follow-up (3)
Quality of life	Health-related quality of life, measured by Short Form Health Survey-12^47^	12	Oral administration of questionnaire with participant	At baseline, after the interventions and after 2 months follow-up (3)
Experience of care	Autonomy support, measured by Health Care Climate Questionnaire^54 56^	6	Written questionnaire for participants*	Mid-intervention (1)
	Patient satisfaction, measured by modified Net Promoter Scale ^53 54^	2	Written questionnaire for participants*	Mid-intervention (1)
	Qualitative assessment focus group		Focus group with participants	After the intervention (1)
Shared decision-making	Observing Patient Involvement in decision making 5 items questionnaire^56^	5	Qualitative assessment by researcher scoring the audio recording of multidisciplinary meeting	Audio-recording of the first multidisciplinary meeting (1)
Administration of positive health methodology	Qualitative assessment		Qualitative assessment by researcher of audio recording of intake consultation	Audio-recording of the first multidisciplinary meeting (1)
Care providers’ perception of intervention	Acceptability of intervention measure, intervention appropriateness measure, feasibility of intervention measure^57^	12	Written questionnaire for care professionals	Before and after the intervention (2)
Integration of care	Integration meter^58^	20	Written questionnaire for care professionals	Before and after the intervention (2)

Oral administration is standard for longer questionnaires or those on complex topics.

* The written questionnaires for participants are sent out by EDC Castor or per post, but oral completion of these questionnaires is offered at the request of the participant or when the questionnaire remains unanswered. The timing of participants’ questionnaires depends on randomisation (see figure 1).

### Primary outcome

#### Cost-effectiveness using cost of care and quality-adjusted life-years

Incremental cost-effectiveness will be based on mean quality of life and mean costs of care from a societal perspective, comparing between intervention and usual care. The validated European Quality of Life 5 Dimensions 5 Level questionnaire (EQ-5D-5L) assesses quality of life, consisting of five items and a visual analogue scale, with a higher score correlating with better quality of life.^41 42^ Cost of care from societal perspective is determined by medical consumption, or care delivered, and productivity (loss). This also includes consultations that are part of the intervention. Costs of care and productivity (loss) are calculated using relevant items, selected per expert opinion, from the IMTA productivity costs questionnaire (iPCQ),^43 44^ the IMTA medical consumption questionnaire (iMCQ)^45^ and the TiC-P questionnaire on healthcare consumption and productivity losses for patients with psychiatric disorders. ^46^ Only data that cannot be reliably retrieved—using the Statistics Netherlands microdata database or the primary care EHR—will be gathered using the eight-item *Care and Work* questionnaire. The *Care and Work* and EQ-5D-5L questionnaires will be sent to all participants as per schedule (figure 1). Data sets for the cost-effectiveness analysis will be linked at the individual level and analysed in a secure environment at Statistics Netherlands, where the pseudonymised data set will be enriched with microdata. Under certain conditions, these microdata are accessible for statistical and scientific research, and Statistics Netherlands can link data sets under strict disclosure conditions in accordance with the General Data Protection Regulation.^47^

### Secondary outcomes

The secondary outcomes consist of psychological outcomes, assessment of positive health methodology and implementation and process evaluation. Three types of psychological outcome measures are included. First, we will assess whether the intervention affects aspects of *self-regulation* using measures of proactive coping, patient activation, self-efficacy and intention. Second, we assess the *experience of care* using measures of patient satisfaction and autonomy-supportive care. Finally, *quality of life* will be assessed to determine whether changes in the aforementioned measures translate into improved well-being. The self-regulation and quality of life measures will be assessed during a face-to-face interview at the start of the intervention, and at 12 and 14 months later. Questionnaires on the experience of care will be administered mid-intervention (figure 1).

#### *Self-regulation*: using measures of proactive coping, patient activation, self-efficacy and intention

The Utrecht Proactive Coping Competence scale is a validated 21-item questionnaire that measures self-rated competence on proactive coping, with 4-point scales, ranging from 1 (not good) to 4 (very good).^48^ Patient Activation Measure-13, a validated 13-item questionnaire, assesses self-reported knowledge, skills and confidence in health management on 4-point scales, ranging from 1 (totally disagree) to 4 (totally agree).^49^ Action self-efficacy (“I am confident in my abilities to [ …]”) and maintenance self-efficacy (“I am confident in my abilities to [….] when encountering obstacles”)^50^ will be assessed in the self-efficacy and intention item list for eight items using 5-point scales, ranging from 1 (totally disagree) to 5 (totally agree). This addresses four key behaviours selected for the purpose of this study (adequate self-care, maintaining daily structure, discussing concerns with care professionals, asking for help in a timely manner). Behavioural execution and intention to perform will also be assessed for each behaviour.

#### *Experience of care*: using measures of patient satisfaction, autonomy-supportive care and qualitative evaluation

The Health Care Climate Questionnaire (HCCQ) and modified Net Promoter scale (mNPS) will be used to assess the experience of care. The HCCQ measures patients’ perceived degree of autonomy support by healthcare providers based on six items assessed on 5-point scales, ranging from 1 (totally disagree) to 5 (totally agree).^51 52^ The mNPS consists of two patient satisfaction-related items, with scores ranging from 1 (worst possible) to 10 (best possible).^53 54^ Additional rich information on patients’ care experiences will be gathered during a focus group meeting at the end of the intervention.

#### Health-related quality of life

Both the EQ-5D-5L and the Short Form Health Survey (SF-12) measure quality of life. The EQ-5D-5L is used in cost-effectiveness calculations, and SF-12 provides self-reported health as a secondary outcome. The SF-12, a 12-item validated questionnaire, measures quality of life across eight dimensions of health using varying scales.^55^

#### Administering positive health methodology, including shared decision-making

With consent of healthcare professionals and participants, the intake consultation and the first multidisciplinary meeting will be audio-recorded to allow qualitative analysis of positive health methodology use.^28^ Themes, conversation techniques and the positive health topics addressed will be evaluated. Shared decision-making will be scored by two independent observers using OPTION5, an observational questionnaire ranging from 0 (not observed) to 5 (high level of shared decision-making).^56^

#### Implementation outcomes, integration of care and process evaluation

Care providers’ perceptions of the proposed intervention will be assessed using the validated acceptability of intervention measure (AIM), intervention appropriateness measure (IAM) and feasibility of intervention measure (FIM),^57^ as well as one item on perceived effectiveness of the intervention. These measures will be sent to contributing care professionals at baseline and upon completion of the intervention. Care integration among care professionals is scored using the *integration meter*, a 20-item questionnaire that scores integration levels for six domains, with scores ranging from 1 (low) to 4 (high).^58^ This questionnaire is to be completed by care professionals pre-intervention and post-intervention.

Data on recruitment and population reach will be gathered during participant inclusion. Process evaluation following the RE-AIM (Reach, Effectiveness, Adoption, Implementation, and Maintenance) framework^59^ will be simultaneous, thus providing insight on practicalities for future implementation.

### Statistical analysis

The effect of offering the Hotspotters Projects’ intervention will be analysed on an intention-to-treat basis, complemented by a per-protocol analysis to assess intervention effects. This will involve a multilevel generalised linear model, with ‘individual’ as the first level, ‘cluster’ as the second and ‘time-to-intervention’ as the third level, with results stratified by gender, and adjustment for time from start of study (continuous; with an interaction between cluster and time, providing each cluster with its own underlying time trend) and total time in the intervention (continuous, with time before intervention as zero). Patient subgroups will also be explored as possible independent variables, based on affected life domains or ICPC codes, neighbourhood and the level of care integration.

#### Cost-effectiveness analysis

The economic evaluation will consist of a cost-utility analysis (cost per quality-adjusted life-year (QALY)) based on the study results, and a cost-utility analysis with a lifetime horizon (cost per QALY), both performed from a societal perspective. In the cost-utility analysis, the mean effects and mean costs during the intervention period will be compared with the preintervention period using two-sided bootstrapping. In a net-benefit analysis, costs will be related to the outcomes and presented as a cost-effectiveness acceptability curve. In the model-based cost-utility analysis, a deterministic decision analysis model will be developed to extrapolate the observed outcomes to lifetime costs and QALYs. Model inputs will be based on literature data and expert opinion. Extensive sensitivity analyses will be performed on the model inputs to get an overall picture of possible costs and QALYs over the remaining life span. Costs will be discounted at a percentage of 4% and effects at a percentage of 1.5%, in accordance with the Dutch guidelines for health economic research.^60^ Sensitivity analyses will be carried out for the most important input parameters.

#### Cost analysis

Intervention costs will be assessed using a micro-costing approach, with detailed data collected on resource use (personnel time, material, equipment). Data on healthcare use will be derived from GP registries and patient questionnaires, information on productivity losses from patient questionnaires. Healthcare use and intersectoral costs will be valued at standard prices published in the Dutch costing guidelines.^61 62^ Costs of absenteeism from paid work will be calculated using the friction cost method.

#### Patient outcome analysis: QALYs

QALYs will be estimated using the EQ-5D-5L, and utilities calculated from the EQ-5D-5L using Dutch tariffs.^63^ During the follow-up period, QALYs will be calculated with an area under the curve method, and utility values entered into a lifetime cost-utility analysis model.

#### Self-regulation and self-reported health

Self-regulation and self-reported health outcomes will be gathered pre-intervention and post-intervention to assess intervention effects and analysed using frequentist statistics, with accounting for regression to the mean if necessary. The data analysis plan will be preregistered prior to data inspection via *OSF* or as *predicted*.

#### Experience of care

Quantitative HCCQ and mNPS data will be analysed descriptively, and focus group meetings will be recorded and transcribed. Thematic content analysis will then be used to create an initial coding book that will be further analysed to arrive at the final coding book.

#### Administering positive health methodology, including shared decision-making

We will use a mixed-analysis approach with transcripts of the recorded intake consultation and first multidisciplinary meeting: using descriptive analysis for shared decision-making outcomes from OPTION5^56^; and both deductive and inductive analyses will be employed to assess the use of positive health methodology, the importance of discussed life domains, patients’ needs, problems and wishes, and how the personalised care plan was formed.

#### Implementation and process evaluation

In addition to descriptive implementation data on recruitment and population reach, pre-post analysis will be used to explore questionnaires on FIM, IAM and AIM, and scores correlated with perceived effectiveness. Level of care integration will also be analysed as a possible independent variable in cost-effectiveness.

## Ethics and dissemination

The ethics committee of Leiden University Medical Centre granted approval for this study (p21.123). Future publications will refer to this protocol and explicitly mention amendments, which will be published in the trial register. This study will be submitted to trial auditing. Results will be shared through peer-reviewed scientific journals and (inter)national conference presentations.

## Discussion

Building on promising intervention strategies,^8 15 20 30^ we expect our primary care-based intervention to address the diverse care needs and improve overall care for Hotspotters. Compared with standard care, the intervention is likely cost-effective from a societal perspective, and we foresee a possible shift in cost from the medical to social domain. Furthermore, by focusing on patients experiencing disruption in several life domains, we anticipate that our approach will better suit the complex and diverse needs of this population.

### Methodological challenges

Previous studies have struggled with regression to the mean in a population defined by high care use,^20^,^22^ often leading to inconclusive results. A strength of our plan is the use of a stepped wedge cluster RCT design, which includes a preintervention control period of varying length (2–8 months). Care use and costs of care are expected to show a regression to the mean effect, which is easier to observe in participants over longer control periods. By comparing outcomes on *time from start of study* and *total intervention time*, we can assess and correct for this effect. Another methodological challenge is the heterogeneity of the proposed study population, as Hotspotters are not defined by a particular diagnosis, complicating the comparison of study participants. However, by combining a stepped wedge cluster RCT design with multilevel analysis, effect comparisons can be made at both the individual and cluster levels.

### Selection bias and risk of drop-out

Selection bias and loss to follow-up are potentially problematic when studying Hotspotters. Random factors that influence study participation are likely and may include low trust,^1^ little social support,^64 65^ low (health) literacy,^66^ as well as multiple health issues that may hinder study participation. We observed a lower inclusion rate in our second pilot, where patients chose to opt out despite having previously expressed interest in participating to their GP practice. After evaluating this process with the involved care providers, the standard study information leaflets proved too long and complex for this population. To minimise possible selection bias, we reformatted all written communications for low literacy, provided video-formatted study information and have limited questionnaires in number, length and frequency (questionnaires may be completed by research staff where necessary). Regarding the anticipated high risk of drop-out, the ensured partaking in the novel intervention may motivate participants to (continue) study participation. This is expected to lower drop-out in the control phase, especially compared with a classic two-armed design, and thus improve control data.

### Perspective on outcomes

‘Hotspotters’ may have persistent health issues that cannot be resolved or reversed by a 1-year intervention.^22^ Nevertheless, based on our first pilot study that showed improved perceived health within 12 months (*unpublished*), we expect the study duration to be sufficient to allow reliable estimates of intervention cost-effectiveness compared with standard care. We envisage that an integrated, personalised approach will promote appropriate care, leading to better perceived health, better care and a possible reduction in acute care encounters. The benefits in cost terms of transitioning from mismatched to appropriate care are still unknown, but we expect a shift from medical to social costs. Nevertheless, appropriate care might entail additional costs, which may be acceptable if accompanied by health gains and/or future savings.

### Recruitment of primary care practices

GPs have a heavy workload, leading to an understandable reluctance to participate in research. Due to intake consultations and multidisciplinary meetings, our study will require a relatively large time investment that might discourage participation of some practices, especially those already facing high demand and the complex care issues often found in disadvantaged neighbourhoods. The GP practices from our pilot studies (one and three practices, respectively) were very much intrinsically motivated in care focused on complex or vulnerable patients without any research experience. For this RCT, we recruited other practices through affiliated healthcare organisations and through our network of GP practices. To facilitate participation, we will offer logistical support to all practices regarding study tasks such as patient selection, as well as arrange additional funding to compensate the additional time commitment.

### Network requirements

This intervention requires primary care practices to collaborate with mental health nurses and social workers. Establishing such collaborations within local structures increases the chance of successful implementation^9^ and contributes to sustainable partnerships that may benefit many other patients. Local teams will make arrangements concerning scheduling, locations, task distribution and patient record keeping. As physical, mental and social care are currently the responsibility of different care organisations and budgets, the transition from reactive ‘care as usual’ to a personalised, integrated approach will require changes at the system, organisational and financial levels. In the case of this study, arrangements concerning time invested, costs and financing have been made with primary care practices, social work organisations, local government, mental healthcare organisations, healthcare insurers and funders of programmes in disadvantaged neighbourhoods. These barriers should be considered in future implementation.

Hotspotters badly need appropriate and scientifically substantiated interventions that promote their health, due to and despite their complex problems. Our study takes these characteristics, and success and failures of previous studies, into account. Care appropriate for Hotspotters will impact health, healthcare, patient experiences and costs. Results are expected in 2027.

## Supplementary material

10.1136/bmjopen-2024-087940 online supplemental file 1

## Data Availability

No data are available.
