# Supplementary material for: Hotspotters Project: a study protocol for a stepped wedge cluster RCT on the cost-effectiveness of 12-month proactive, integrated and personalised care for patients with problems on multiple life domains and high acute care use
Source: BMJ Open. 2025 Aug 10;15(8):e087940. doi: 10.1136/bmjopen-2024-087940 (PMC12336530; doi:10.1136/bmjopen-2024-087940)
Supplement: online supplemental file 1 [file bmjopen-15-8-s001.pdf]

Consent Form

- **I agree to receive additional appointments at the GP practice.**
- **I agree to complete questionnaires.**
- **I agree that the researcher may access my medical records at the GP to count the amount of care I needed.**
- **I agree that the researcher may access my medical records at the GP to see which illnesses I had.**
- **I agree that the researcher may request information from the CBS on how often I needed care.**
- **I agree to the audio recording of two conversations.**
- **I understand that the audio recording is not a requirement for participation in the study.**
- **I understand the purpose of this study.**
- **I know that my name will not be mentioned, and no one will know that I participated in this study.**
- **I know that I can withdraw from the study at any time.**
- **I understand that the group interview will be recorded on a tape recorder, but only the researcher will listen to it.**

Name participant____

Date and location___________________________________

Signature participant__________________________

Name researcher______________________________

Date and location___________________________________

Signature researcher___________________

Consent form Hotspotters Project v5, dd 25-07-2024. This consent form has been translated into English for the publication of the study protocol.
