# Supplementary material for: Hotspotters Project: a study protocol for a stepped wedge cluster RCT on the cost-effectiveness of 12-month proactive, integrated and personalised care for patients with problems on multiple life domains and high acute care use
Source: BMJ Open. 2025 Aug 10;15(8):e087940. doi: 10.1136/bmjopen-2024-087940 (PMC12336530; doi:10.1136/bmjopen-2024-087940)
Supplement: Supplementary file 2 [file bmjopen-2024-087940.reviewer_comments.pdf]

PEER REVIEW HISTORY

BMJ Open publishes all reviews undertaken for accepted manuscripts. Reviewers are asked to complete a checklist review form and are provided with free text boxes to elaborate on their assessment. These free text comments are reproduced below.

ARTICLE DETAILS

Title (Provisional)

The Hotspotters Project: a study protocol for a stepped wedge cluster RCT on the cost-effectiveness of 12 months proactive, integrated and personalised care for patients with problems on multiple life domains and high acute care use

Authors

Tiemes, Vera; Leming, Kimberley A; Borgdorff, Hanneke; Bruijnzeels, Marc Abraham; van Gestel, Laurens C; Adriaanse, Marieke A; van der Wel, Mark; van den Akker-van Marle, Elske M; Numans, Mattijs E.; Vos, Rimke C

VERSION 1 - REVIEW

**Reviewer**	**1**
**Name**	**Dhungana, Raja Ram**
**Affiliation**	**International Organization for Migration Nepal, Health**
**Date**	**29-Jul-2024**
**COI**	**none**

---

none

**Reviewer**	**2**
**Name**	**De Nooijer , Kim**
**Affiliation**	**Vrije Universiteit Brussel**
**Date**	**08-Aug-2024**
**COI**	**None.**

This is a very interesting and important study on the cost-effectiveness of 12 months proactive, integrated and personalized care for patients with problems in multiple life domains and high acute care use. The following points would enhance the study.

Introduction:

First paragraph: Could the authors explain more about who “hotspotters” are? Is this term used in other studies as well? And could the authors give examples of the problems these patients experience in the different life domains?

Second paragraph: the authors refer to other pilot studies, however, it is not clear to me what these interventions entail? And in which settings are these interventions implemented? Are there already RCTs conducted focusing on ‘hotspotters’ or only pilot studies?

Methods:

Study design: randomization will be performed by researchers involved in the current study? Or those independent from the study? Could the authors elaborate on this.

Sample size calculation: the power analysis was based on a loss to follow-up of 20%, is this 20% based on a previous, similar, RCT? Can the authors provide a reference for this?

Study population: Why do the researcher or research support centre not physically meet the patient for the informed consent procedure? Are those conducting the recruitment procedure different from those conducting the outcome measurements?

Intervention: What is the role of the informal care provider in the intervention? Are their care needs, as both care recipient and care provider, discussed and addressed as well?

Measurements: By whom are the outcomes measured? Why did the authors choose to administer them via post and not physically (like the recruitment procedure)?

Measurements: I would suggest reporting all the outcomes, measurement instruments, number of items per instrument etc. in a Table so that it is easier to read what has been measured, by whom, how etc.

Measurements: Will the authors also collect information regarding what has been delivered to the patient, for instance number of multidisciplinary meetings that have been organized per patient, who attended these meetings, what has been discussed etc.

Statistical analysis: Where and when will the data analysis plan be published?

Discussion: As the authors state in the discussion, recruitment of primary care practices might be difficult. Can the authors explain how well recruitment went in the pilot study? What did the authors change in the recruitment strategy of the full scale RCT? And what are the experiences of the authors with the recruitment of patients?

**Reviewer**	**3**
**Name**	**Manguro, Griffins**
**Affiliation**	**Ghent University**
**Date**	**03-Feb-2025**

COI None

Very well written paper. I like that it is simple to understand.

It will be very important for you to explain, in greater detail, the origin of the word hotspotter. You have provided some explanation in the text, but as this is not a universally known term, please explain further to avoid confusion.

**Reviewer**	**4**
**Name**	**Kuwornu, Paul**
**Affiliation**	**Queensland University of Technology**
**Date**	**27-Feb-2025**
**COI**	**None**

The manuscript is generally good. I have attached specific comments for the author's consideration.

The authors have proposed an important intervention for a high-need group of patients. The following suggestions are provided for the authors to consider in strengthening the manuscript:

1. Typo on page 2 line 60 – "risk of" not "risk op" the fourth bullet point of the article summary.
2. ""Hotspotters" – patients characterized by problems in multiple life domains and high acute care use". This definition/characterization appears to define this group based on both needs and healthcare use. This initial characterization/definition contrasts with the one later proposed by the authors focusing purely on "disturbed life domains". It will be important to clarify which perspective (needs-based vs. consequence) the authors adopt. If the statement is made with regard to the general characterization of this population in the literature, some references may need to be provided.
3. The authors commented on their first pilot study and later stated, "Building on our pilot studies". Can the authors clarify how many pilot studies were done and what was learnt from them to inform the current study?
4. The authors stated that "Clusters – as defined by a team of primary care physician(s) and a mental healthcare nurse cooperating in the same practice population – will be to one of four groups to determine the start of intervention". Can the authors clarify who the other three groups are? Is this in reference to Figure 1? This needs clarifying.
5. "Recruitment will alternate between practice and algorithm lists to ensure a 50/50 split, and patients found on both lists will be first approached". It is unclear from this description whether patients were told they were being approached because an ACG -based algorithm identified them for the study.

6. “The intervention consists of a positive health or 4D intake with goal setting”. Can the authors clarify why these two “intervention approaches” were used? Does the mental healthcare nurse decide which one to use? Does the choice differ by patient type?
7. “Incremental cost -effectiveness will be based on comparing quality of life and societal cost between (at least) two interventions”. It is unclear how many interventions there are in the study. Could the author also clarify whether they will be comparing intervention periods vs. different interventions?
8. “In the cost -utility analysis, the effects and costs during the intervention period will be compared to the pre -intervention period using two -sided bootstrapping.” It will be important for the authors to clarify what statistic is being compared. Is it the mean, median, or other summary measure of the effect/costs?
9. “In the model-based cost -utility analysis, a model will be developed to extrapolate the observed outcomes to lifetime costs and QALYs.” What kind of model will this be? Markov model? Given there are different ways of doing this extrapolation, some preferable over others, it will be better for the authors to clarify their intended approach.
10. Are patient transportation costs going to be captured in the cost-utility analysis?

VERSION 1 - AUTHOR RESPONSE

Reviewer 2 question 1: First paragraph: Could the authors explain more about who “hotspotters” are? Is this term used in other studies as well? And could the authors give examples of the problems these patients experience in the different life domains?
Reply: The term ‘hotspotters’ is based on Gawande’s landmark article on “Hot Spotting”, which describes that accumulation of medical problems concentrate geographically. This article resulted in increased attention from researchers and policy makers for patients groups based on costs or use of care, previously referred to as high-cost patients, super-utilizers, frequent- attenders or High-Costs, high-need patients. Our definition of ‘hotspotters’ builds on these concepts, but includes a needs-based perspective with ‘problems on multiple life domains’. The first two paragraphs of the introduction have been rewritten as follows:
“*This study will test a novel intervention aimed at “Hotspotters” – patients with health problems spanning physical, mental and/or social domains, in combination with high acute care use. The definition of this population is based on Gawande’s landmark article on ‘Hot Spotting’[1], which described that accumulation of medical problems concentrate geographically – in a neighbourhood, street or even a building. This drew attention from policy makers and researchers* for patients groups based on costs or use of care, previously referred *to high-cost patients[2-6], High-Cost High-Need patients[7- 13], super-utilisers of care and frequent attenders of emergency departments[14-22].*
The extensive use of (acute)care suggests that existing care options do not match Hotspotters' care needs. This results in costly[23, 24] and ineffective care, suboptimal health outcomes and a poor patient experience of care[17]. In addition to a heavy but partially avoidable burden on the healthcare system as a whole[3, 4, 18, 23], healthcare professionals may become frustrated and feel they are ‘fighting a losing battle’[1].

This current study defines Hotspotters by disturbed life domains (a needs-based perspective) and high acute care use(current care needs, consequence on care system). Problems on multiple life domains will result in various co-existing conditions, each impacting daily life. Physical problems refer to chronic physical conditions, and mental problems to diagnosis such as depression or substance abuse. By also including problems in the social domain (for example housing, poverty, loneliness etc), we build on the 'Hot Spotting' concept where the geographical clustering points to sociodemographics as a contributing factor."

Reviewer 2 question 2: Second paragraph: the authors refer to other pilot studies, however, it is not clear to me what these interventions entail? And in which settings are these interventions implemented? Are there already RCTs conducted focusing on 'hotspotters' or only pilot studies?
Reply: Thank you for bringing this to our attention. The Hotspotters project is our first cost- effectiveness study focusing on 'Hotspotters'. This study was preceded by two primary care based pilot studies. The first pilot study was a case series that evaluated whether the positive health interview and tailored welfare support could be considered suitable care for the problems that Hotspotters face. The second pilot was a feasibility study for the current intervention study, including the selection of patients using an algorithm and invitation for study participation using standard study information leaflets. The manuscript is revised to clearly indicate which pilot study is mentioned. Examples of what tailored support in the first pilot entails have been added:

"*In our first pilot study, 25 patients with high acute care use and problems across multiple life domains received an extensive Positive Health interview[27, 28] at their GP practice, followed by tailored welfare-support such as housing or finding volunteer work. The mean positive* health score improved by 22%, from 5.5 (1.5SD) to 6.7 (1.1SD), at one year (unpublished)."
"*Our second pilot, a feasibility study in primary care, was conducted in preparation for this study(unpublished). Its lessons are reflected in the current design and implementation. Building on our two pilot studies, we now aim to evaluate the cost- effectiveness of a 12-month proactive, personalised care approach for Hotspotters that integrates physical, mental and social care."*

"*In our second pilot, a feasibility study(unpublished), the standard study information leaflets proved too long and complex for this population, leading to frequent opt-outs amongst potential participants."*

“*Nevertheless, based on our first pilot study that showed improved perceived health within 12 months (unpublished), we expect the study duration to be sufficient to allow reliable estimates of intervention cost-effectiveness compared to standard care.”*

‘Patient feedback from both pilot studies is incorporated in the design of this study and a separate qualitative study is performed regarding patient care preferences.”

Reviewer 2 question 3: Study design: randomization will be performed by researchers involved in the current study? Or those independent from the study? Could the authors elaborate on this.
Reply: Randomisation will be performed by two researchers involved in the current study. A track record is kept of each randomisation step for monitoring purpose. The manuscript is complemented on this topic:
“*Randomisation will be performed centrally by two researchers (VT and RV) using computer-generated random numbers and will be announced after recruitment*.”

Reviewer 2 question 4: Sample size calculation: the power analysis was based on a loss to follow-up of 20%, is this 20% based on a previous, similar, RCT? Can the authors provide a reference for this?
Reply: As this study focusses on a rather new population there is no literature on expected drop-out rate among *Hotspotters*. The sample size calculation was made in close collaboration with a statistician, using the clinical outcomes of the first pilot study and regional costs of care data of the defined group. After consulting with both statistician and clinical experts on
Hotspotter-populations, we decided to increase the expected drop-out rate from the commonly used 10% to 20% to ensure adequate power.

Reviewer 2 question 5: Study population: Why do the researcher or research support centre not physically meet the patient for the informed consent procedure? Are those conducting the recruitment procedure different from those conducting the outcome measurements?
Reply: First, patients are contacted by their GP practice with the question whether they would like more information about this study. After receiving the study information leaflet, patients receive a phone call from a researcher. During this conversation, there is ample opportunity for questions and, if there is interest in participation, it is discussed what taking part in the study involves. After that, they can either make an informed decision on participation, or request additional time to consider. This procedure complies with ethical guidelines.
We had experience with informed consent procedures per phone calls with our second pilot, as it was not possible to meet them due to COVID-19 restrictions. The informed consent procedure per telephone allowed us to be more flexible in setting an appointment with

potential participants. Next to that, it prevented patients from having to travel to the practice once more, and it proved to be more practical logistically for the participating GP practices as they often had limited rooms available to conduct these conversations.

Reviewer 2 question 6: Intervention: What is the role of the informal care provider in the intervention? Are their care needs, as both care recipient and care provider, discussed and addressed as well?
Reply: Informal care providers can play an important role for patients with complex care needs, however this study focusses on the patients themselves. While the needs of informal care providers may be discussed during the intake or multi-disciplinary meeting, it is not a standard topic. The intervention takes place at the primary care practice where the patient is registered, which brings the advantage of a care team that already has intimate knowledge of a patients situation and whether they have an informal care provider or not.

Reviewer 2 question 7: Measurements: By whom are the outcomes measured? Why did the authors choose to administer them via post and not physically (like the recruitment procedure)?
Reply: A table of all outcomes is now included in the manuscript, describing per outcome the measurement instrument(s) used, number of items per instrument, frequency and method of administration. The questionnaires have been carefully selected based on the intended outcome, complexity of questions and length. Only for longer questionnaires or those on more complex topics we decided for oral administration as a standard. Oral administration requires additional time investment from participants, and given the study's extended duration, we wanted to be mindful of this. However, oral administration is available for all questionnaires based on participants' preference or is offered if a questionnaire remains unanswered. The option for oral administration of questionnaires can be found in the manuscript under "measurements";
"All written questionnaires will be sent per email or post, or may be administered per phone interview either on request or when a questionnaire is incomplete."

Reviewer 2 question 8: Measurements: I would suggest reporting all the outcomes, measurement instruments, number of items per instrument etc. in a Table so that it is easier to read what has been measured, by whom, how etc.
Reply: Thank you for this suggestion. A table of all outcomes is now included in the manuscript (*see also the reply to previous question*).

Reviewer 2 question 9: Measurements: Will the authors also collect information regarding what has been delivered to the patient, for instance number of multidisciplinary meetings that have been organized per patient, who attended these meetings, what has been discussed etc.

Reply: Information on care delivered to patients is included into the costs calculations. This includes information on number of consultations, multidisciplinary meetings and who attended these meetings. The intake consultation and the multidisciplinary meetings are audio-recorded (*if all who are present in the meeting consent to recording)*, which are used for qualitative analysis on shared decision making and administration of the positive health methodology.

Reviewer 2 question 10: Statistical analysis: Where and when will the data analysis plan be published?
Reply: The data analysis plan for the outcomes on self-regulation and self-reported health will be pre-registered before the end of the study.

Reviewer 2 question 11: Discussion: As the authors state in the discussion, recruitment of primary care practices might be difficult. Can the authors explain how well recruitment went in the pilot study? What did the authors change in the recruitment strategy of the full scale RCT? And what are the experiences of the authors with the recruitment of patients?
Reply: The first pilot study took place in 1 healthcare centre. The second pilot took place at three GP practices which had shared personal and had a pre-established close collaboration. For both pilots, the practices were involved in care innovations and clinical studies focussed on complex or vulnerable patients. For this RCT, we recruited practices through affiliated healthcare organisations and through our network of GP practices.
The experiences of participant recruitment during our second pilot led us to modify the study information leaflet. The leaflet was adapted for length and low literacy, and was made available in video-format. Recruitment experiences of this RCT will be evaluated in a process evaluation.

Reviewer 3 Question 1: Very well written paper. I like that it is simple to understand. It will be very important for you to explain, in greater detail, the origin of the word hotspotter. You have provided some explanation in the text, but as this is not a universally known term, please explain further to avoid confusion.
Reply: Thank you for this comment. The first two paragraphs of the introduction are rewritten to better describe the origin of the Hotspotter-population. Please see the reply to the first question of reviewer 2.

Reviewer 4 comment 1: Typo on page 2 line 60 – "risk of" not "risk op" the fourth bullet point
of the article summary.
Reply: Thank you for bringing this typo to our attention. It has been corrected.

Reviewer 4 comment 2: ""Hotspotters" – Reviewer 4 comment 2: ""Hotspotters" – patients characterized by problems in multiple life domains and high acute care use". This definition/characterization appears to define this group based on both needs and healthcare

use. This initial characterization/definition contrasts with the one later proposed by the authors focusing purely on "disturbed life domains". It will be important to clarify which perspective (needs based vs. consequence) the authors adopt. If the statement is made with regard to the general characterization of this population in the literature, some references may need to be provided.

Reply: We believe that the needs-based perspective is a valuable addition to existing population definitions. In our view, this cannot be separated from care use, as it is, after all, an expression of current care needs. Thus, 'high care use' can be seen from a needs-perspective as well as a consequence for the care system. Therefore we focus on the subgroup of patients with complex problems that have both problems on multiple life domains and high acute care use. In the revised manuscript, we elaborated and nuanced the argumentation for our definition and perspective on Hotspotters (*see the revised first 2 paragraphs of introduction)*

Reviewer 4 comment 3: The authors commented on their first pilot study and later stated, "Building on our pilot studies". Can the authors clarify how many pilot studies were done and what was learnt from them to inform the current study?

Reply: We would like to refer to our reply of **Reviewer 2 question 2.**

Reviewer 4 comment 4: The authors stated that "Clusters – as defined by a team of primary care physician(s) and a mental healthcare nurse cooperating in the same practice population – will be to one of four groups to determine the start of intervention" Can the authors clarify who the other three groups are? Is this in reference to Figure 1? This needs clarifying.

Reply: We revised the paragraph on randomization of clusters for clarity. Clusters are randomized to determine the duration of the control period – either 2, 4, 6, or 8 months. The term "groups" added unnecessary confusion and is no longer used in this paragraph or in the accompanying figure 1.

Reviewer 4 comment 5: "Recruitment will alternate between practice and algorithm lists to ensure a 50/50 split, and patients found on both lists will be first approached". It is unclear from this description whether patients were told they were being approached because an ACG -based algorithm identified them for the study.

Reply: All patients that are invited for study participation fit the study inclusion- and exclusion criteria, regardless of the selection method. As the selection method has no further effect on the intervention, it is not discussed with patients. This is information is added to the manuscript:
"*The selection method has no impact on the intervention, and therefore not discussed with participants*."

Reviewer 4 comment 6: "The intervention consists of a positive health or 4D intake with goal setting". Can the authors clarify why these two "intervention approaches" were used? Does the mental healthcare nurse decide which one to use? Does the choice differ by patient type?

Reply: This is clarified under *"formal training".* The intake in the intervention uses a semi- structured conversation tool on perceived health in multiple domains. "Therefore, we offered formal training in the positive health method, which is currently the most used method in the Netherlands. However, if the mental healthcare nurse is already formally trained in the used of the 4D-model, this may be used instead.

Reviewer 4 comment 7: "Incremental cost -effectiveness will be based on comparing quality of life and societal cost between (at least) two interventions". It is unclear how many interventions there are in the study. Could the author also clarify whether they will be comparing intervention periods vs. different interventions?

Reply: This study compares 1 intervention to standard care, which is measured during the control period. This sentence is rephrased to better reflect this: "Incremental cost-effectiveness will be based on comparing mean quality of life and societal cost between intervention and usual care".

Reviewer 4 comment 8: "In the cost -utility analysis, the effects and costs during the intervention period will be compared to the pre -intervention period using two -sided bootstrapping." It will be important for the authors to clarify what statistic is being compared. Is it the mean, median, or other summary measure of the effect/costs?

Reply: The mean outcomes on effects and costs will be compared between the intervention period and usual care. This is now included in the manuscript as follows:

"*Incremental cost-effectiveness will be based on comparing mean quality of life and societal cost between intervention and usual care*".

"*In the cost-utility analysis, the mean effects and mean costs during the intervention period will be compared to the pre-intervention period using two-sided bootstrapping."*

Reviewer 4 comment 9: "In the model-based cost -utility analysis, a model will be developed to extrapolate the observed outcomes to lifetime costs and QALYs." What kind of model will this be? Markov model? Given there are different ways of doing this extrapolation, some preferable over others, it will be better for the authors to clarify their intended approach.

Reply: Since the extrapolation is intended to give an overall picture of costs and QALYs over the rest of the life span, we will use a rather simple deterministic decision analysis model to extrapolate the observed outcomes to costs and QALYs over the rest of the life span. Model inputs will be based on literature data and expert opinion. Extensive sensitivity analyses will be performed on the model inputs to get a picture of the range of lifetime outcomes. The manuscript is adapted as follows:
"In the model-based cost -utility analysis, a deterministic decision analysis model will be developed to extrapolate the observed outcomes to lifetime costs and QALYs. Model inputs will be based on literature data and expert opinion. Extensive sensitivity analyses will be performed on the model inputs to get an overall picture of possible costs and QALYs over the remaining life span."

Reviewer 4 comment 10: Are patient transportation costs going to be captured in the cost- utility analysis?

Reply: Transportation costs will not be captured in the cost-utility analysis. Participants will have additional appointments at their primary care practice. Patients are registered with a primary care practice close to their home, with (in the Netherlands) negligible travel time and costs.

VERSION 2 - REVIEW

**Reviewer**	**4**
**Name**	**Kuwornu, Paul**
**Affiliation**	**Queensland University of Technology**
**Date**	**14-May-2025**
**COI**	

The authors have addressed the reviewer comments, and the manuscript has improved.

VERSION 2 - AUTHOR RESPONSE

Reviewer: 4 Dr. Paul Kuwornu, Queensland University of Technology

Comments to the Author: The authors have addressed the reviewer comments, and the manuscript has improved.

Reply: Thank you very much for reviewing our manuscript and thereby contributing to the improvement of this work.

Reviewer: 4 If you have selected ‘Yes’ above, please provide details of any competing interests.:

Not applicable

Reply: If we did not understand this comment correctly please let us know, but there are no competing interests to declare.
